# A framework for group-wise summarization and comparison of chromatin state annotations

**DOI:** 10.1093/bioinformatics/btac722

**Published:** 2022-11-07

**Authors:** Ha Vu, Zane Koch, Petko Fiziev, Jason Ernst

**Affiliations:** Bioinformatics Interdepartmental Program, University of California, Los Angeles, Los Angeles, CA 90095, USA; Department of Biological Chemistry, University of California, Los Angeles, Los Angeles, CA 90095, USA; Department of Biological Chemistry, University of California, Los Angeles, Los Angeles, CA 90095, USA; Bioinformatics Interdepartmental Program, University of California, Los Angeles, Los Angeles, CA 90095, USA; Department of Biological Chemistry, University of California, Los Angeles, Los Angeles, CA 90095, USA; Illumina Artificial Intelligence Laboratory, Illumina Inc., Foster City, CA 94404, USA; Bioinformatics Interdepartmental Program, University of California, Los Angeles, Los Angeles, CA 90095, USA; Department of Biological Chemistry, University of California, Los Angeles, Los Angeles, CA 90095, USA; Eli and Edythe Broad Center of Regenerative Medicine and Stem Cell Research, University of California, Los Angeles, Los Angeles, CA 90095, USA; Computer Science Department, University of California, Los Angeles, Los Angeles, CA 90095, USA; Jonsson Comprehensive Cancer Center, University of California, Los Angeles, Los Angeles, CA 90095, USA; Molecular Biology Institute, University of California, Los Angeles, Los Angeles, CA 90095, USA; Computational Medicine Department, University of California, Los Angeles, Los Angeles, CA 90095, USA

## Abstract

**Motivation:**

Genome-wide maps of epigenetic modifications are powerful resources for non-coding genome annotation. Maps of multiple epigenetics marks have been integrated into cell or tissue type-specific chromatin state annotations for many cell or tissue types. With the increasing availability of multiple chromatin state maps for biologically similar samples, there is a need for methods that can effectively summarize the information about chromatin state annotations within groups of samples and identify differences across groups of samples at a high resolution.

**Results:**

We developed CSREP, which takes as input chromatin state annotations for a group of samples. CSREP then probabilistically estimates the state at each genomic position and derives a representative chromatin state map for the group. CSREP uses an ensemble of multi-class logistic regression classifiers that predict the chromatin state assignment of each sample given the state maps from all other samples. The difference in CSREP’s probability assignments for the two groups can be used to identify genomic locations with differential chromatin state assignments. Using groups of chromatin state maps of a diverse set of cell and tissue types, we demonstrate the advantages of using CSREP to summarize chromatin state maps and identify biologically relevant differences between groups at a high resolution.

**Availability and implementation:**

The CSREP source code and generated data are available at http://github.com/ernstlab/csrep.

**Supplementary information:**

Supplementary data are available at *Bioinformatics* online.

## 1 Introduction

Genome-wide maps of chromatin marks such as histone modifications and variants provide valuable information for annotating non-coding genome features (Barski *et al.*, 2007; Ernst *et al.*, 2011; Xie *et al.*, 2013; Zhu *et al.*, 2013). Efforts by large consortia and individual labs have produced chromatin state maps for many cell and tissue types (ENCODE Project Consortium, 2012; Roadmap Epigenomics Consortium *et al.*, 2015; Xie *et al.*, 2013; Zhu *et al.*, 2013). A popular representation of such data is chromatin states defined by the combinatorial and spatial patterns of multiple marks, which are generated by methods such as ChromHMM and Segway (Ernst and Kellis, 2010, 2012; Hoffman *et al.*, 2012; Libbrecht *et al.*, 2021), and correspond to diverse classes of genomic elements including various types of enhancers and promoters.

Chromatin state maps have been produced for hundreds of different biological samples. In many cases, there are multiple samples representing similar cell and tissue types (Boix *et al.*, 2021; Roadmap Epigenomics Consortium *et al.*, 2015). In such cases, to simplify analyses and visualizations, it may be desirable to have a single chromatin state annotation that summarizes the annotations for all samples in a pre-defined sample group of interest. A straightforward approach to this task is to take the most frequent chromatin state assigned at each position across samples in the group. However, when the number of samples in a group is small or the number of states is large, such an approach can be particularly vulnerable to noise. Furthermore, such an approach does not consider additional information available about the different chromatin states. For example, if a location was assigned to three different states in three samples, the summary annotation among these three states based on the frequency-based method would be arbitrary. However, by leveraging information about the co-occurrence of state assignments genome-wide, there is additional information to predict the most likely chromatin state annotation for a new sample from the group.

A related challenge is to identify differences in chromatin state annotations between two groups at a high resolution and on a per-state basis. Several methods have been developed for comparing chromatin state annotations between groups of samples, but typically either work at a coarse resolution or do not identify differences on a per-chromatin-state basis. For instance, ChromDiff (Yen and Kellis, 2015) presents a statistical testing framework to uncover pre-defined broad regions such as gene bodies with significant differences for specific chromatin states across the two groups, but was not specifically designed for detecting differences at the resolution of the chromatin state annotations. EpiAlign (Ge *et al.*, 2019) scores the alignment patterns between two user-input sequences of chromatin state annotations in two samples, hence is most applicable for comparing broad domains that encompass multiple chromatin state segments. Another method, chromswitch (Jessa and Kleinman, 2018) also offers a framework to score the differential chromatin state annotations within broader user-specified input genomic locus and is not designed for detecting chromatin state differences genome-wide at the same resolution of the annotations. EpiCompare (He and Wang, 2017) is primarily a webtool that can be used for detecting cell-type-specific chromatin state differences in terms of enhancer or promoter states but does not support detecting differences for individual states or other types of chromatin states. SCIDDO (Ebert and Schulz, 2021) conducts fast genome-wide detection of differential chromatin domains between two groups of samples while incorporating a measure of similarity among states. However, as SCIDDO provides a single differential score per position, it does not directly answer the question of which chromatin states change at each genomic position. Another method, dPCA (Ji *et al.*, 2013), works directly on chromatin mark signals and does not quantify state differences across groups of samples.

To effectively summarize the chromatin state annotations for a group of samples and prioritize the chromatin state differences between two groups on a per-state basis, at high resolution, we introduce CSREP. CSREP leverages both the information about the input samples’ chromatin states at a position, as well as information on states’ co-occurrences in different samples within the same group across the genome. CSREP does this by first generating probabilistic estimates of chromatin state annotations to summarize a group of samples using an ensemble of multi-class logistic regression classifiers. These classifiers predict the state assignment in a sample at a position, given the annotations in other samples at the corresponding genomic position. From those predictions, CSREP is then able to produce a single summary state assignment per position. Furthermore, CSREP can use the difference of summary probabilistic predictions for two groups of samples to quantify the difference in state assignments between the two groups on a per-state basis, e.g. one genome-wide score track per chromatin state. CSREP’s ability to summarize chromatin states for a group of samples beyond simple counting is a unique feature of CSREP relative to existing methods mentioned above for detecting differential chromatin states or domains. CSREP is also distinguished from these existing methods by a combination of (i) considering differential chromatin state annotations at the resolution of the input annotations instead of over broad domains, (ii) generating outputs genome-wide instead of at user-specified loci and (iii) providing state-specific and directionally meaningful scores for all states.

Using CSREP, we generate the summary chromatin state maps for 11 groups of tissue/cell types from Roadmap Epigenomics Project (Roadmap Epigenomics Consortium *et al.*, 2015), and for 75 groups from the EpiMap Portal (Boix *et al.*, 2021), which can be easily viewed on genome browsers (Data availability). We show that CSREP can better predict chromatin state assignments in held-out samples than a counting-based baseline method. We also verify that the resulting summary chromatin maps show correspondence with the group’s average gene expression profile. Additionally, we show that CSREP’s differential scores can recover differential epigenetic signals on chromosome X between Male and Female samples. We also show that CSREP differential scores between samples from two different tissue groups can predict regions of differential peaks for various chromatin marks. The CSREP implementation is designed to be user-friendly and includes a detailed tutorial, available at https://github.com/ernstlab/csrep. We expect CSREP will be a useful tool for summarizing chromatin state maps within groups and finding differences across groups. Additionally, we expect the summary annotations for different tissue groups that we generated with CSREP to be a useful resource.

## 2 Materials and methods

### 2.1 CSREP’s summarization of a group of samples

Let G denote the number of genomic bins across the genome, S the number of chromatin states, and N the number of samples in the target group of samples. Let $C_{i,n}\;$denote the chromatin state assigned to sample n at genomic position i, which can take one value of 1, 2, …, S. Let $N_{n}$ denote the set of samples not including n, i.e. $N_{n}=\left\{1,\ldots ,N\right\}-\left\{n\right\}$. In general, CSREP is an ensemble of N multi-class logistic regression classifiers such that for each sample n, CSREP trains a classifier to predict the chromatin state map of this sample based on features from the remaining samples ($N_{n}$). The predictor variables for such a model include one-hot encoded chromatin state maps of the N-1 samples (all samples in the group except n) and an intercept term, resulting in $\left(N-1\right)\times S+1$ predictor variables. The response variable is the chromatin state of the target sample n, which can take one value of 1, 2, …, S.

In the multi-class logistic regression model, let $X_{i}\;$denote the vector of predictor variables at position i, which has length $\left(N-1\right)\times S+1$ and takes values {0,1}. The last entry of $X_{i}\;$is 1, corresponding to the intercept term. Let $Y_{i}$ denote the value of the response variable at position i, which takes values {1,2,…,S}. Since the input chromatin state maps that we used segmented the genome into 200-bp bins, we refer to each genomic position as one 200-bp window in the genome. We randomly selected genomic positions for the training dataset, such that these positions constitute 10% of the genome. We chose 10% as the training proportion because increasing this parameter does not result in considerable increase in model accuracy but increases runtime (Supplementary Fig. S1). Given the training dataset, for each state s∈{1,…,S-1}, the multi-class logistic regression model learns a coefficient vector $\beta_{s}$ with length $\left(N-1\right)\times S+1$, corresponding to the number of predictor variables. The probability of sample n’s chromatin state assignment being s at position i is then calculated as:

$$P\left(Y_{i}=s\right)=\;\frac{e^{\beta_{s}\times X_{i}}}{1+\;\sum_{j=1}^{S-1}e^{\beta_{j}\times X_{i}}}$$

for s∈{1, ., S-1}, and as the following when s=S*:*

$$P\left(Y_{i}=S\right)=\;\frac{1}{1+\;\sum_{j=1}^{S-1}e^{\beta_{j}\times X_{i}}}.$$

The model is implemented using Python’s sklearn, pybedtools packages and snakemake (Dale *et al.*, 2011; Köster and Rahmann, 2012; Mölder *et al.*, 2021; Quinlan and Hall, 2010). An L2-norm penalty with the default regularization strength of 1.0 was used for training. CSREP applies the model to generate probabilistic predictions of a genome-wide chromatin state map for sample n, which is presented in a matrix of size G×S. The output matrices from N predictions for N samples are then averaged, so at each genomic bin, the sum of state assignment probabilities across S states is 1. In addition, the chromatin state with the maximum probability in each row is recorded to produce a single representative chromatin state map for the entire group of samples.

### 2.2 CSREP’s application to prioritizing differential chromatin state changes between two groups of samples

To calculate differential chromatin state maps between two groups of samples, Group1 and Group2, CSREP first calculates the probabilistic chromatin state map matrices for each group as described above, denoted as $R_{1}$ and $R_{2}$, respectively. After this, CSREP subtracts the two matrices to represent the differential chromatin state map between Group1 and Group2 (denoted $D_{12}$), i.e. $D_{12}\;=\;R_{1}\;-\;R_{2}$. We note that we used signed and not absolute difference here and thus the score ranges from −1 to 1. A score on row i and column s of $D_{12}$, denoted $D_{12,i,s}$, being −1 means Group2 is estimated to have probability 1 of being assigned to state s at position i while Group1 has probability of 0. Additionally, since CSREP assigns S scores of differential chromatin maps to each genomic position i, corresponding to S states, CSREP can uncover specific chromatin state changes. For example, if $D_{12,i,s}=0.8$ when s=1 while $D_{12,i,s}=\;-0.8$ when s=2, we can infer that at position i, Group1 is likely to be in State 1 while Group2 is likely to be in State 2.

## 3 Results

### 3.1 CSREP method overview

**Fig. 1.**
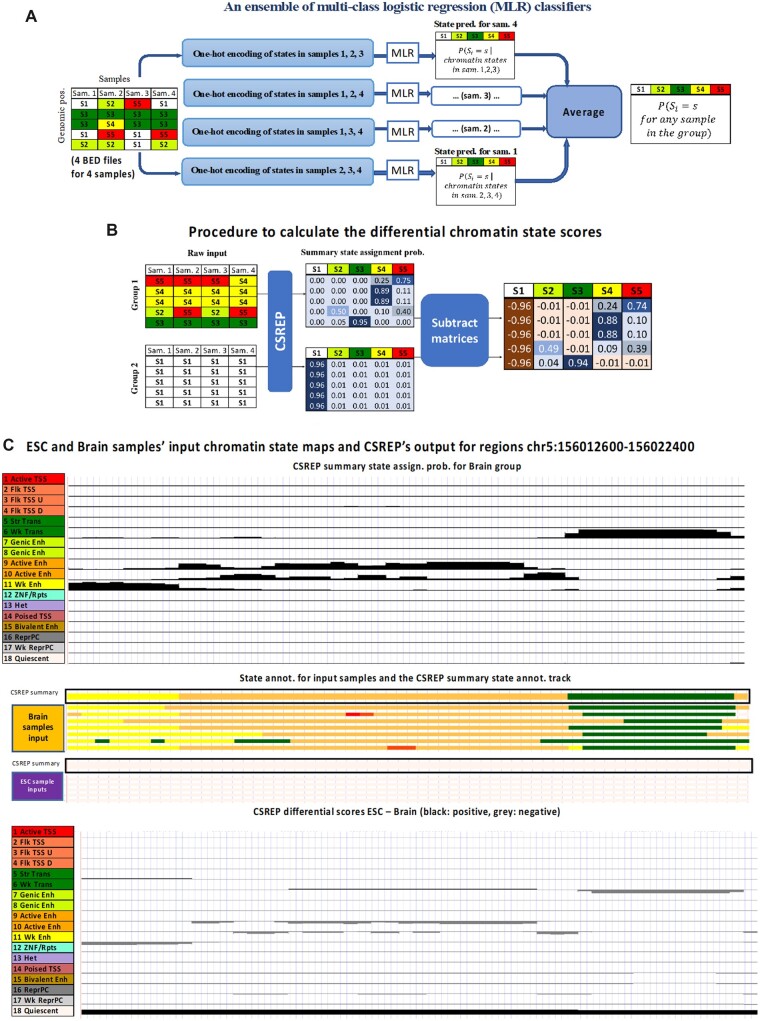
Overview of CSREP. (**A**) CSREP uses an ensemble of multi-class logistic regression models. In each model, the chromatin state map at the target sample is predicted based on the one-hot encoding of chromatin state assignments at the corresponding genomic positions in other samples. Multi-class logistic regression outputs the probabilities that each genomic position (row) in the target sample will be assigned to each state (column). CSREP averages the prediction matrices for target samples, to output the summary state assignment probability matrix. Sam., sample; $P\left(S_{i}=s\right),$ probability that genomic position i is annotated as state s. (**B**) The operations to obtain differential chromatin state scores between two groups with multiple samples. CSREP calculates the summary chromatin state assignment matrices for two groups and then subtracts one group’s summary matrix from the other’s to obtain differential chromatin scores. Differential chromatin scores are bounded between −1 and 1. (**C**) Visualization of CSREP’s output in a genomic region (hg19, chr5:156012600–156022400). The top of the subpanel shows the CSREP’s summary chromatin state probabilities for 18 states across 7 Brain reference epigenomes. Each track shows the probabilities of assignment for one state, as named and colored on the left. The middle subpanel shows the 18-state chromatin state maps for 7 Brain samples and 5 ESC samples from Roadmap Epigenomics (Roadmap Epigenomics Consortium *et al.*, 2015), and the CSREP’s output summary chromatin state maps for each group, boxed. States are colored as in legends at the left of this subpanel. The last subpanel shows the differential chromatin scores when Brain’s summary state probabilities are subtracted from ESC’s (ESC—Brain). Each track shows one state’s differential scores. Scores between 0 and 1 are shown above each track, while those between −1 and 0 are below the corresponding track. This region is also shown in an expanded format in Supplementary Figure S2

CSREP takes as input chromatin state maps for a group of samples learned in such a way that annotations for different samples have an internally consistent set of defined chromatin states (Ernst and Kellis, 2010, 2012). We note that the input is presented in BED file format, with each file containing the chromatin state map for one sample. CSREP then generates as output (i) a summary probabilistic chromatin state assignment matrix and (ii) a summary state map track for the group. The summary state assignment matrix represents the probabilities of each state being present at each genomic position in a new sample of that group. To generate these, CSREP takes a supervised learning approach, leveraging information about the co-occurrence of states from the different samples across the genome. Specifically, for each group of input samples, CSREP trains an ensemble of N multi-class logistic regression classifiers (Hastie *et al.*, 2009), where N is the number of samples in the group, to generate probabilistic predictions for each chromatin state at each position (Fig. 1A, Section 2). We used multi-class logistic regression classifiers since they provide well-calibrated probabilities, are robust, and relatively fast to train. Each classifier is trained with *labels* based on the chromatin state assignments from one sample and *features* based on the chromatin state assignments in other samples for the same genomic positions. Each classifier then makes a probabilistic prediction of the chromatin state assigned at each genomic position in the target sample. The chromatin state input features to each logistic regression classifier are represented with a one-hot-encoding of the chromatin states. The classifiers are trained on randomly selected genomic positions that constitute 10% of the genome, while the predictions are calculated genome-wide. The resolution of predictions is the same as that of input samples’ chromatin state maps (200 bp with default settings for ChromHMM). The prediction results for each sample’s chromatin state map are represented in a matrix with *rows* corresponding to genomic positions and *columns* chromatin states. The values in each row, which sum to 1, represent the probabilities of state assignments at a genomic position. The probabilistic summary of a group is based on averaging the prediction output matrices for each sample in the group. These probabilistic predictions are then used to generate a summary chromatin state map for the group of samples by assigning the state with maximum assignment probability to each genomic position (Fig. 1A, Section 2).

CSREP’s summary probabilistic predictions can be directly used to generate differential chromatin state maps for two groups with multiple samples, where the input samples from both groups share the same internally consistent set of defined chromatin states. This is achieved by subtracting the summary chromatin state assignment matrix of one group (first group) from the other’s (second group) (Fig. 1B, Section 2). At each genomic position, CSREP’s chromatin differential scores for individual chromatin states are bounded between −1 and 1. A score of 1 for state s means state s was predicted to be the annotation for the first and second groups with probability 1 and 0, respectively, and vice versa for −1 (Fig. 1B and C, Supplementary Fig. S2). Overall, in addition to summarizing the state assignments for groups of samples, CSREP can calculate scores of differential chromatin state assignments for pairs of groups at the resolution of the input chromatin state maps.

**Fig. 2.**
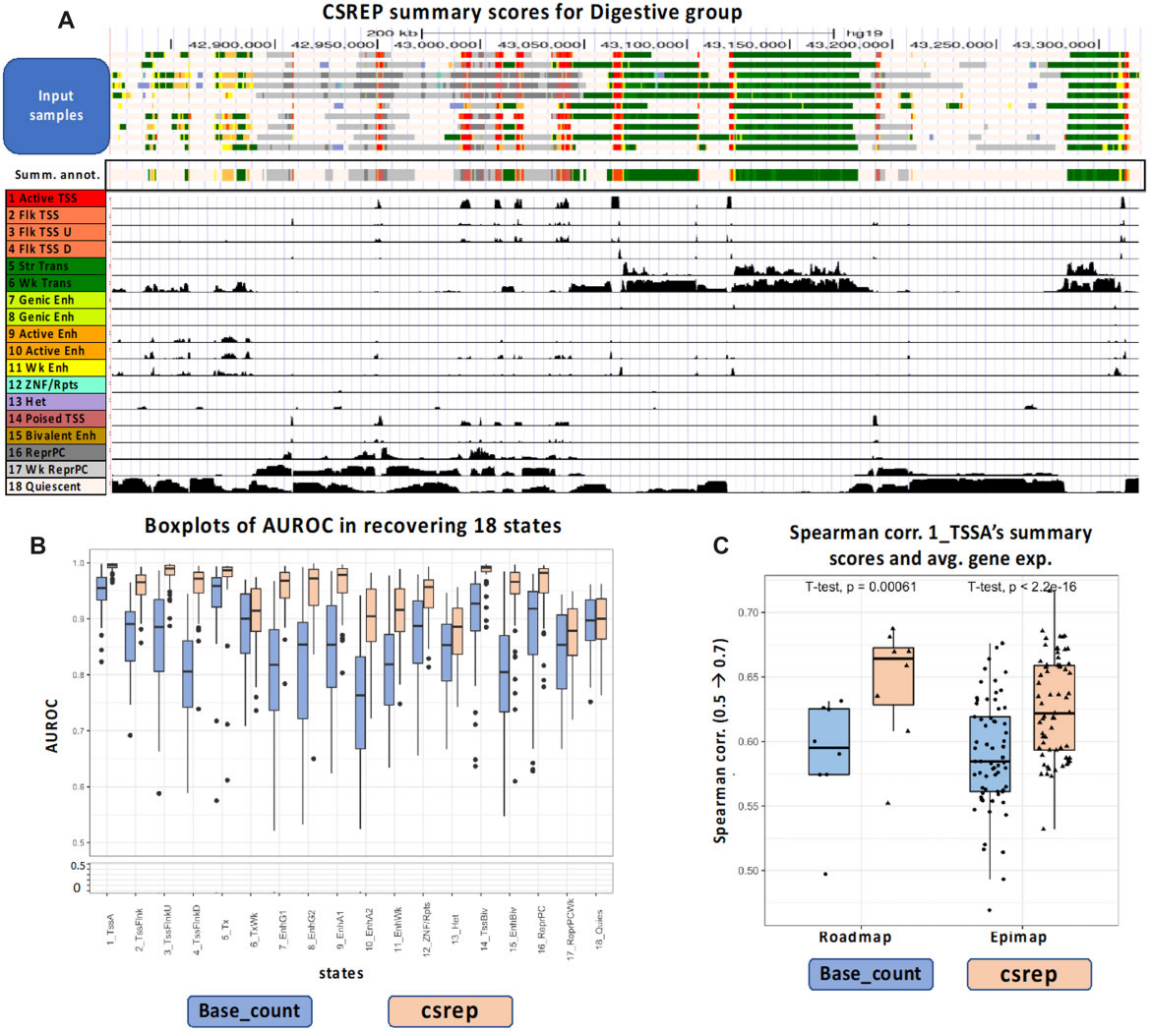
Performance of CSREP in summarizing multiple samples’ chromatin state maps from a group. (**A**) Visualization of one arbitrarily selected 500-kb region (chr5: 42821109–43321109, hg19). The first 10 tracks show chromatin state maps of 10 samples of the Digestive group from the Roadmap Epigenomics Consortium, which were input to CSREP. The following track shows the summary chromatin state map from CSREP, which shows strong agreement with the input. States are colored based on the legend on the lower left. In the following 18 tracks, each track shows CSREP’s probabilities of assignment for each of 18 states, with the state annotations shown in the legend on left. (**B**) Boxplots showing the CSREP and base_count methods’ average, range and 25, 75% quantiles of the AUROCs across 64 samples, for each of the 18 chromatin states. The AUROCs were calculated in leave-one-out cross-validation analysis where we used a group’s summary probabilistic chromatin state map to predict genomic locations of individual chromatin states in a left-out sample from the same cell/tissue group (Supplementary Methods). States 1–18 (x-axis) are annotated as in (A). (**C**) Boxplots showing the Spearman correlations between a group of samples’ (1) summary probabilities of state 1_TssA (active TSS) at annotated TSSs and (2) the corresponding group’s average gene expression (Supplementary Methods). We obtained the correlations for 8 groups of cell types from the Roadmap Epigenomics Project and 65 groups from EpiMap. Each dot shows the Spearman correlation for data from a group of samples. Results of paired *t*-test to compare CSREP versus base_count’s output correlations are shown on top. The alternative hypothesis for the *t*-test is that correlations resulted from CSREP are higher than those from base_count (Supplementary Methods)

### 3.2 CSREP is predictive of chromatin states on held-out samples

We applied CSREP to a compendium of 18-state chromatin state maps for 64 samples (reference epigenomes) from 11 tissue groups generated by the Roadmap Epigenomics Project (Roadmap Epigenomics Consortium *et al.*, 2015). The tissue groups include embryonic stem cells (ESCs), induced pluripotent stem cells (iPSC), ESC-derived cells, blood & T-cells, HSC & B-cells, epithelial, brain, muscle, heart, smooth muscle and digestive. The number of input samples for each tissue group ranges from 3 to 12 (Additional File 2). We provide CSREP’s genome-wide summary probabilistic and state assignments for the 11 tissue groups (Data availability). Given our computing configuration, the run-time for CSREP to jointly preprocess input data for all 64 samples was ∼40 min, and then the time to output the predictions for each group ranged from ∼1 to 3 h (Supplementary Fig. S3, Supplementary Methods).

We first visualized CSREP’s summary chromatin state maps for groups of samples from digestive and heart tissue groups, which have 10 and 3 samples, respectively (Fig. 2A, Supplementary Figs S4–S7). We arbitrarily selected four 500-kb regions and for each group, we visualized the input chromatin state maps and CSREP’s output probabilistic state estimates and summary state map at such genomic windows. We observed expected correspondences between the groups’ input and output chromatin state assignment estimates (Fig. 2A, Supplementary Figs S4–S7). We also visualized CSREP’s summary chromatin state maps at the loci of two genes that had distinctly higher expression in Digestive and Brain cell types, LGALS4 and MT3, respectively, which highlighted the corresponding groups’ differences in the summary chromatin state maps (GTEx Consortium, 2020) (Supplementary Figs S8–S10).

To quantitatively evaluate CSREP’s summary output for a group of samples, we evaluated the accuracy of CSREP’s summary probabilistic chromatin state predictions in a leave-one-out cross-validation analysis. In particular, for each chromatin state, we calculated the area under the receiver operating characteristic curve (AUROC) for predicting genomic locations assigned to the state in a held-out sample, based on the summary chromatin state maps generated from data in other samples from the group (Supplementary Methods). We compared the performance of CSREP against a baseline method, denoted base_count (short for counting-based baseline method), which counts each state’s frequency across input samples at each genomic position (Supplementary Methods).

CSREP showed strong predictive performance for chromatin states in left-out samples with average AUROCs across 64 samples varying from 0.871 to 0.993 for the 18 states. Across the 18 states, CSREP consistently had better AUROC in recovering individual states compared to the baseline method base_count (Fig. 2B). The average AUROC improvement by CSREP compared to base_count ranged from 0.003 (for state 18_Quies) to 0.157 (for state 4_TssFlnkD). Larger performance improvements by CSREP relative to base_count were observed for all chromatin states when there are fewer input samples in the group (Supplementary Fig. S11).

### 3.3 CSREP’s summary chromatin state maps’ association with gene expression

Transcription start sites (TSS) are marked by various histone modifications and variants that can correlate with transcription (Kimura, 2013; Soboleva *et al.*, 2014). Here, we evaluated how CSREP’s summary state map for a tissue group is predictive of the group’s gene expression profiles at the TSS of genes. First, we obtained gene expression data for available samples for the 11 tissue groups mentioned above and calculated the average protein-coding gene expression for each group (Supplementary Methods). Of the 11 groups, 8 had gene expression data available for at least one sample (Supplementary Methods). We then calculated the Spearman correlation between (i) the group’s average expression for protein-coding genes and (ii) CSREP’s summary state assignment probabilities for state 1_TssA (active TSS state) at the corresponding genes’ TSSs. We did the same evaluation for base_count. CSREP had significantly higher correlations than base_count (Fig. 2C, paired *t*-test *P*-value < 0.0062, average 0.65 versus 0.59, Supplementary Methods). We next extended this analysis for a larger dataset of 552 samples in 75 groups from EpiMap repository based on state 1_TssA from the same 18-state annotations (Boix *et al.*, 2021) (Supplementary Methods). The 75 groups were previously formed based on tissue types and developmental stages with the number of samples per group ranging from 3 to 38 (Supplementary Methods, Additional File 2). Of the 75 groups, 65 also had gene expression data available for at least one sample. Across these 65 groups, again CSREP had significantly higher correlations than base_count (Fig. 2C, paired *t*-test *P*-value < 2.2e-16, average 0.63 versus 0.59, Supplementary Methods). Overall, CSREP’s summary chromatin state maps at TSS for the TssA state show significantly higher correspondence with gene expression levels compared to the base_count method.

### 3.4 CSREP detects differential chromatin regions associated with different sexes

We next investigated the performance of CSREP at identifying biologically meaningful chromatin state changes between groups of Male and Female samples based on its ability to prioritize chromatin state differences on chromosome X (chrX) relative to autosomal chromosomes. Specifically, we applied CSREP to calculate differential chromatin state scores between 25 Female and 44 Male samples from Roadmap Epigenomics (Supplementary Methods) (Ge *et al.*, 2019; Yen and Kellis, 2015) by subtracting CSREP’s summary state probability matrix for the Female samples from the corresponding matrix for the Male samples.

**Fig. 3.**
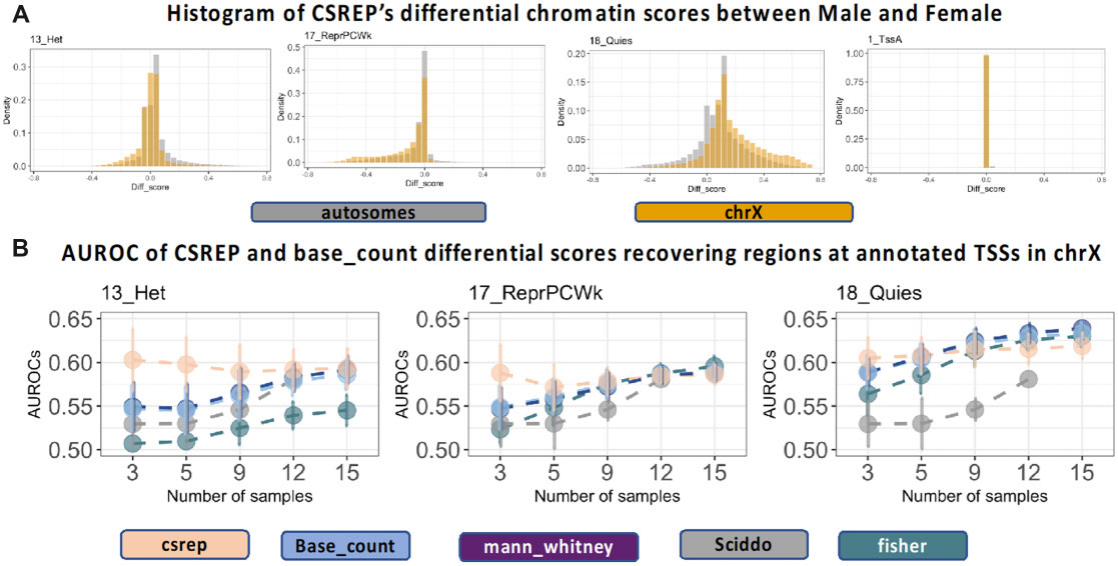
CSREP shows signals of differential chromatin state scores in chrX when comparing Male and Female samples. (**A**) Each subpanel shows the histogram of CSREP’s differential scores in autosomes and chrX, for states associated with heterochromatin (13_Het), weak polycomb repressed domains (17_ReprPCWk), quiescent regions (18_Quies), and active transcription start site (1_TssA). The x-axis shows differential scores, with positive values implying Male samples have higher probabilities of being in the state compared to Female samples, and vice versa for negative values. Histograms of scores for all states are in Supplementary Figure S12. (**B**) AUROCs of recovering regions overlapping annotated TSSs on chrX, using differential chromatin scores of three states as in (A), outputted by CSREP, base_count, SCIDDO, Mann–Whitney *U*-test (based on ChromDiff) and Fisher’s Exact test (based on EpiCompare) for Male and Female groups (Supplementary Methods). The AUROCs based on Mann–Whitney *U*-test showed values close to those based on base_count, hence the plotted average AUROCs from these two methods were overlapping. We calculated the AUROCs using different sets of input Male and Female samples, with varying numbers of samples in each group (x-axis). For each number of samples (x-axis), we conducted the analysis for 30 sets of Male and Female input samples (Supplementary Methods). The plots show the average (dots) and standard deviation (error bars) of the AUROCs across the 30 sets of input samples. SCIDDO did not successfully generate output for the case of 15 input samples, thus no results are reported for that

We analyzed CSREP’s differential scores for all chromatin states across autosomal chromosomes and chrX (Fig. 3A, Supplementary Figs S12 and S13). Three states with the largest magnitude of difference in mean Male–Female differential scores between chrX and autosomes were states 13_Het (heterochromatin, marked by H3K9me3), 17_ReprPCWk (weak polycomb repressive complex) and 18_Quies (quiescent) (Supplementary Fig. S13). In contrast, active promoter/enhancer states showed minimal difference in the distribution of Male–Female differential scores for chrX versus autosomes (Fig. 3A, Supplementary Figs S12 and S13). In chrX, compared to autosomal chromosomes, the distribution of differential scores for states 13_Het and 17_ReprPCWk showed a larger tail of negative values. ChrX’s average score minus the autosomes’ average score values for states 13_Het and 17_ReprPCWk were −0.039 and −0.054, respectively (Supplementary Fig. S13), implying that on chrX, Female samples are more often assigned to these states compared to Male samples. State 18_Quies showed the opposite trend with a difference of 0.11 (Fig. 3A, Supplementary Fig. S13). These results are consistent with sex-specific chrX inactivation, which is used in Female mammals to achieve dosage compensation between the two sexes (Wutz, 2011; Yen and Kellis, 2015).

We next compared the performance of CSREP and other methods in recovering annotated transcription start sites (TSSs) on chrX, using the above-mentioned states, given varying numbers of input samples (Supplementary Methods, Fig. 3B). To do this, we randomly selected 30 subsets of size n Male and n Female samples from the set of available 44 Male and 25 Female samples, where n is varied within the set of 3, 5, 9, 12 or 15 samples. Given each set of input Male and Female samples, we calculated the AUROC when using differential chromatin scores between Male and Female groups to predict locations overlapping annotated TSSs on chrX, against the background of those overlapping all annotated TSSs in the genome (Supplementary Methods). The methods we compared CSREP against include SCIDDO, the count difference from base_count, the Mann–Whitney *U*-test [used by ChromDiff (Yen and Kellis, 2015)], and the Fisher’s exact test [used by EpiCompare (He and Wang, 2017)] (Supplementary Methods). The Mann–Whitney *U* and Fisher’s exact tests were applied at each genomic position, using two input sample groups’ chromatin state annotations at the respective position. We considered other related methods for detecting differential chromatin domains not appropriate for direct comparison against CSREP (Supplementary Methods). We observed that CSREP showed the largest advantage over other methods, as measured by AUROCs, when the number of input samples from Male and Female groups is relatively small, e.g. three samples in each group (Fig. 3B). As the number of input samples from each group increases sufficiently, the overall performance advantage of CSREP relative to base_count, Mann–Whitney *U*-test and Fisher’s exact test goes away. In all cases, CSREP showed better performance compared to SCIDDO (Ebert and Schulz, 2021) (Fig. 3B). Overall, CSREP showed the clearest advantage over other approaches when the number of samples is relatively small, which occurs frequently in practice.

### 3.5 CSREP’s differential scores recover differential chromatin mark peaks

We next analyzed how well CSREP’s differential chromatin state scores can predict genomic regions overlapping differential signals of DNase I hypersensitivity (DNase), H3K9ac and H3K27ac between samples from embryonic stem cell (ESC) and brain. DNase and H3K9ac signals were not used for learning the 18-state model used to annotate the two groups’ input samples, providing an independent validation. While H3K27ac was used in learning the input chromatin state maps, since all the methods being compared (CSREP, base_count, SCIDDO, Mann–Whitney *U*-test based on ChromDiff and Fisher’s exact test based on EpiCompare) had access to the same chromatin state maps as input, and H3K27ac is a well-established mark of cell-type-specific activity (Creyghton *et al.*, 2010), we still considered H3K27ac in the evaluations of methods’ performance.

For each of the three chromatin marks, we first obtained a set of bases that overlap with peaks in all samples from ESC but not in any from the Brain group and vice versa (Supplementary Methods, Additional File 2). We then calculated CSREP and base_count differential chromatin scores by subtracting the summary chromatin state map of Brain from that of the ESC. Additionally, we applied SCIDDO, Mann–Whitney *U*-test (ChromDiff’s approach) and Fisher’s Exact test (EpiCompare’s approach) to the same set of input data (Supplementary Methods). We evaluated, in terms of AUROC, how well the methods prioritize regions overlapping bases in the ESC-/brain-specific sets of peaks (Supplementary Methods). For CSREP, base_count, Mann–Whitney *U*-test and Fisher’s exact test, we conducted separate evaluations for each chromatin state but did *not* for SCIDDO since it outputs one score track that measures the overall difference across the chromatin state landscape between the two groups.

**Fig. 4.**
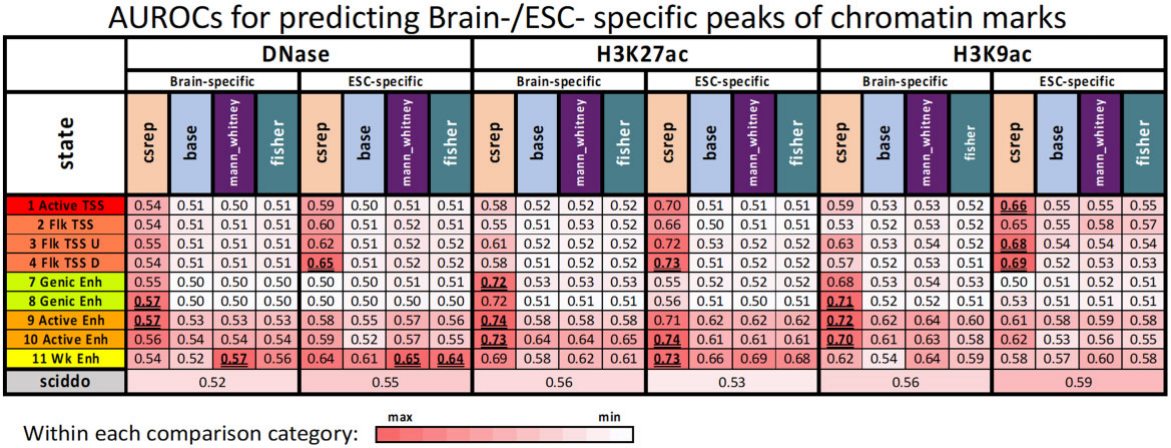
Evaluation of recovery of differential chromatin marks signals between ESC and Brain. The table shows AUROCs for differential scores’ predictions of genomic regions associated with differential peak signals for one chromatin mark, from left to right: DNase, H3K27ac and H3K9ac. For each chromatin mark, it shows the AUROCs of predicting signal peaks observed in Brain and ESC exclusively (Brain-spec and ESC-spec, respectively). Differential scores outputted by CSREP, base-count, Mann–Whitney *U*-test (used by ChromDiff) and Fisher’s exact test (used by EpiCompare) are shown for active promoter and enhancer associated chromatin states (rows). In each category of comparisons (a chromatin mark in either ESC or Brain), the top three scores that show the highest AUROCs are in bold and underlined. Along the bottom is the AUROC for SCIDDO. Only active promoter and enhancer states are expected to be associated with differential DNase, H3K27ac and H3K9ac signals, but the AUROCs corresponding to all states are shown in Supplementary Figure S14

Across the different marks and groups (ESC-specific or Brain-specific peaks) we evaluated, CSREP’s differential scores from either promoter- or enhancer-associated states resulted in the highest AUROCs, with few exceptions (Fig. 4, Supplementary Fig. S14). For example, for identifying Brain-specific H3K9ac peaks, CSREP had an AUROC of 0.717 based on the evaluation with state 9_EnhA1, an active enhancer state, while the maximum AUROCs achieved for base_count, Mann–Whitney *U*-test, Fisher’s exact test and SCIDDO were 0.617, 0.636, 0.601 and 0.564, respectively. In total across the six evaluations, among the top-3 highest AUROCs per evaluation, 15 of the 18 AUROCs were based on CSREP’s differential scores for individual chromatin states (Fig. 4). The AUROCs for states not usually associated with these marks (transcription, heterochromatin, repeats/ZNF gene, quiescent and polycomb repressed states) tended to be near 0.5 or in some cases lower (Supplementary Fig. S14). These analyses suggest that CSREP differential scores tended to better correspond to locations of individual mark differences between two groups of samples genome-wide, compared to other approaches. Even though SCIDDO incorporated a measure of dissimilarity among states, it showed lower AUROCs compared to the maximum obtained by CSREP. This is potentially because SCIDDO outputs one score per genomic bin to measure the general difference across all states, while CSREP generates state-specific scores. Hence, CSREP should have better power to predict regions associated with differential signals of marks that are present in only specific states (e.g. H3K27ac is present in enhancer states but not in repressive states). Additionally, this may also be because CSREP produces scores that show the direction of differences (with positive/negative scores implying one group’s higher state assignment probabilities compared to the other’s) while SCIDDO’s scores do not have a specific direction associated with them.

## 4 Discussion

Here, we proposed CSREP, a method for probabilistically summarizing the chromatin state maps from a group of samples. CSREP achieves this by training multi-class logistic regression models to predict the chromatin state annotations of one sample using data from others, and then averaging the prediction probabilities across all samples in the group. CSREP outputs the probabilities of each chromatin state being assigned to each genomic position, at the same resolution that chromatin states are annotated. We applied CSREP to generate summary 18-state chromatin state assignment probability matrices for 11 groups of cell and tissue types from Roadmap Epigenomics Project (Roadmap Epigenomics Consortium *et al.*, 2015), and 75 groups of samples stratified by cell and tissue types and developmental phases from EpiMap (Boix *et al.*, 2021), and have made them publicly available (Data availability, Additional File 2).

Our analyses reveal that CSREP’s probabilistic summary of state assignments better predicts the chromatin states of held-out samples compared to the counting-based baseline approach. We also showed that CSREP’s summary assignment probabilities of state 1_TssA at TSS were well correlated with the average gene expression of the group and significantly higher than those achieved by the counting-based baseline.

CSREP can also be used to directly quantify the difference in chromatin state maps between two groups with multiple samples, at the resolution of the input annotations. CSREP produces differential scores for each chromatin state at each genomic position, which represent the difference in probabilities that samples from the two input groups are assigned to each specific state. Therefore, CSREP differential scores are bounded (−1 to 1), interpretable with respect to specific chromatin state changes, and indicative of the direction of change, which contrasts it with other approaches that provide a single score showing the magnitude of difference per genomic position. We used CSREP to compare the chromatin state annotations between Male and Female samples from Roadmap Epigenomics (Roadmap Epigenomics Consortium *et al.*, 2015) and showed that CSREP can better predict regions overlapping genes’ TSS on chrX, particularly when there are few samples in each group. CSREP’s differential scores for states associated with active enhancers and promoters better recovered tissue-group-specific peaks of DNase, H3K27ac and H3K9ac signals compared to alternative approaches, suggesting that CSREP provides useful additional information for analyzing epigenomic changes across tissue types.

Here, we presented applications of CSREP on samples that were grouped based on cell and tissue types and based on sex. In general, CSREP assumes the dominant signal of any variation between groups is associated with the grouping variable of interest. In cases in which the experimental design used to collect the data cannot ensure this, other known covariates can be used to detect if there are potential confounders.

CSREP works directly off of chromatin state annotations, which makes CSREP agnostic to the specific methods used to produce those annotations. Some methods for learning chromatin state annotations have the option to expose posterior probability estimates of annotations. However, in general, it is not clear how well calibrated those estimates will be, and assuming accurately determined posterior probability estimates are available as input would also make CSREP less generally applicable. A possible direction for future work would be to extend CSREP to make use of posteriors or possibly other information that CSREP does not directly consider, such as the individual mark signal in each sample.

We note that CSREP’s summary chromatin state maps offer complementary benefits to the recently developed universal chromatin state annotation, which provides a single integrative annotation of the genome based on a model defined from over a 1000 epigenomic datasets from over 100 cell and tissue types (denoted the full-stack model) (Vu and Ernst, 2022). The full-stack model jointly captures activity across many diverse cell and tissue types and hence can capture annotations corresponding to both constitutive and cell-type-specific activities (Supplementary Fig. S2). CSREP, on the other hand, provides a more direct and focused chromatin state annotation representative specifically of the individual input samples’ annotations.

To facilitate the use of CSREP, we provide an implementation of CSREP as a snakemake pipeline (Köster and Rahmann, 2012; Mölder *et al.*, 2021) with a detailed tutorial that only requires users to modify parameters in a yaml file. The program can be run either on local computers or on computing clusters, in which case snakemake will optimize the workflow for execution.

We expect CSREP to be a useful tool and the CSREP output we provided to be a valuable resource for summarizing chromatin state maps from groups of samples, and for prioritizing regions with differential chromatin state changes across pairs of groups of samples.

## Supplementary Material

btac722_Supplementary_Data

## Data Availability

The summary chromatin state maps (the chromatin state assignment matrices and the corresponding state annotation) for 11 tissue groups in Roadmap Epigenomics Project and 75 groups in EpiMap Portal are available for download with links from https://github.com/ernstlab/csrep, and can be viewed on UCSC Genome Browser with the track hub link from https://github.com/ernstlab/csrep. The summary state maps for samples in Roadmap Epigenomics and EpiMap are provided for both hg38 and hg19. Links to download all input data are provided in Additional File 2.
