# Supplementary material for: A framework for group-wise summarization and comparison of chromatin state annotations
Source: Bioinformatics. 2022 Nov 7;39(1):btac722. doi: 10.1093/bioinformatics/btac722 (PMC9805555; doi:10.1093/bioinformatics/btac722)
Supplement: btac722_Supplementary_Data [file btac722_supplementary_data.zip › btac722_Supplementary_Data/AF1_supp_materials_for_publication.docx]

**Primary data sources**

We analyzed genome-wide 18-state chromatin state annotation for 64 reference epigenomes from the Roadmap Epigenomic Project Portal (Roadmap Epigenomics Consortium *et al.*, 2015) and 552 from the EpiMap portal (Boix *et al.*, 2021). We will refer to each reference epigenome as a sample. State annotation data for samples from Roadmap Epigenomics and EpiMap were in hg19. The 18-state model was shared between Roadmap Epigenomics and EpiMap, and was trained based on data of 6 chromatin marks: H3K4me1, H3K4me3, H3K27ac, H3K27me3, H3K36me3 and H3K9me3. We assigned 64 samples from Roadmap Epigenomics into 11 groups based on the accompanying metadata’s tissue group labels. These groups include Blood & T-cell, Brain, Digestive, embryonic stem cells (ESC), ES-deriv, Heart, induced pluripotent stem cells (iPSC), Muscle, Epithelial, smooth muscle (Sm_Muscle) and HSC & B-cell. We assigned the biosamples from EpiMap into 75 distinct groups based on the metadata corresponding to unique combination of: extended biosample summary (tissue and sub-tissue types) and life stage (adult or embryonic, any biosamples with samples of unknown life stage filtered out from the analyses). We only analyzed groups of samples from EpiMap with at least 3 biosamples. Among sample groups from Roadmap Epigenomics, the number of samples per group ranged from 3 to 12, while the corresponding range for samples from EpiMap is 3 to 38. Details about the samples’ ID, groups and other metadata are provided in **Additional File 2**.

Using CSREP, we generated summary chromatin state maps for chromosomes 1-22 and X for the 11 groups from Roadmap Epigenomics and 75 groups from EpiMap using input data in hg19. We ran CSREP on a high-performance compute cluster where each job was allocated 4 cores with 4 GB of memory per core. The run-time for CSREP to jointly preprocess input data for all 64 samples from Roadmap Epigenomics was ~40 minutes, and then the time to output the predictions for each group ranged from ~1 to 3 hours (**Supp. Fig. 3, Supp. Methods**)**.** We then used liftOver from the UCSC genome browser to lift the summary state maps for all groups from either Roadmap Epigenomics or EpiMap from hg19 to hg38. This procedure first finds a one-to-one mapping for a subset of 200-bins between hg19 and hg38, i.e. if there are multiple bins from hg19 that got mapped to the same bin in hg38, those bins would not be included into the annotations. Then, we map both the state assignment probabilities and the summary state annotations for each bin in hg19 to the corresponding bin in hg38. Source code for the liftOver procedure, along with a detailed tutorial, is provided at <https://github.com/ernstlab/csrep>.

**Evaluation of CSREP in representing a group’s chromatin state maps**

We evaluated CSREP and an alternative baseline approach called base_count (defined below) for predicting representative chromatin state maps. We conducted this evaluation through a leave-one-out cross validation framework. Given a group with $N$ samples, for each sample indexed $n$, we evaluated the prediction of chromatin state map for sample $n$ when the state maps of the other $N-1$ samples were used as the input for generating the predictions. For these evaluations we used the data for the 64 samples from the 11 tissue groups from the Roadmap Epigenomics Project (Roadmap Epigenomics Consortium *et al.*, 2015) described above.

***Base_count method:*** Let $C_{nis} = 1$ if in sample $n$, at genomic position $i$, the observed chromatin state is $s$, and $C_{nis} = 0$ otherwise. The base_count approach represents the group’s chromatin state map by calculating the frequency of state $s$ being assigned at the $i$position (${BC}_{is}$) across the samples. In particular:

$${BC}_{is} = \frac{\sum_{n = 1}^{N} C_{nis}}{N}$$

where $N$ is the number of samples. Similar to CSREP, the output matrix for base_count method is of size $G \times S$, with the sum of values in each row being 1, where $G$ and $S$ represent the number of genomic bins and the number of states, as explained in the main Methods.

***Calculating the ROC curves of prediction for a single chromatin state’s location:*** In each round of cross-validation, one sample with index $n$ is held-out. We then used CSREP and base_count to get the summary probabilistic chromatin state map for the group using input data from the remaining $N-1$ samples. For each state $s$, CSREP and base_count output the summary probability that each 200-bp genomic bin gets assigned to the state $s$. We divided the $[0,1]$probability range into 500 equal-width windows with lower bounds $l\in\{0, 0.002, ..., 0.998\}$. Within each probability window, any genomic positions with assignment probability for the state $s$ being no less than the window lower bound ($l$) will be predicted as being in state $s$ for sample $n$. Given the true chromatin state map in sample $n$, we then calculated the cumulative true positive rates and false positive rates of the prediction at each probability threshold $l$ to obtain the ROC curve. This analysis is repeated for each chromatin state, resulting in $S$ ROC curves.

***Evaluating CSREP’s summary chromatin state maps’ association with gene expression***

We obtained gene expression data from the Roadmap Epigenomics Consortium (Roadmap Epigenomics Consortium *et al.*, 2015), which was available as a matrix of values in RPKM (reads per kilobase million) for genes in a subset of the samples from

https://egg2.wustl.edu/roadmap/data/byDataType/rna/expression/57epigenomes.RPKM.pc.gz.

We obtained the accompanying gene annotation information from

https://egg2.wustl.edu/roadmap/data/byDataType/rna/expression/Ensembl_v65.Gencode_v10.ENSG.gene_info.gz.

We filtered out genes that were not annotated as protein-coding, then transformed the gene expression matrix by adding a pseudo-count of 1 to the RPKM counts, and finally log-transformed the resulting values. We also only included genes that were on chromosomes 1-22 and X in hg19 for this analysis, resulting in 20,787 distinct TSSs whose associated gene expression was available. For each of the 11 groups of samples in Roadmap Epigenomics, we obtained the group’s average gene expression profile by averaging over the gene expression values across all available samples in the group, i.e. samples that are both in the group and among the samples whose expression data was available. Among the 11 groups, 8 groups (all except for groups HSC & B-cell, iPSC and Sm_Muscle) had available gene expression data from the available samples. We then calculated the Spearman correlation of the summary chromatin state assignment probabilities for the 1_TssA state at positions that overlap with the 20,787 annotated TSSs and their corresponding average gene expression for each group.

For EpiMap, we obtained data of quantile-normalized protein coding genes’ expression from

https://personal.broadinstitute.org/cboix/epimap/rnaseq_data/merged_qn_log2fpkm.pc.mtx.gz,

which is available as values in log2(FPKM), along with data of samples’ ID and genes’ Ensembl ID. We utilized the same genes’ annotation information as provided by Roadmap Epigenomics, and only included protein-coding genes on chromosomes 1-22 and X in hg19. This resulted in 18,542 distinct TSSs whose associated gene expression was available from EpiMap. Among the 75 groups, 10 groups (SMTH.Digestive, HSC.MPP, EYE.EMB, EPTH.BREAST.EPITH, CA.UCEC, CA.RCC, CA.MYELOMA, BRN.EMB.BRN, BRN.CAUD.NUC, BONE.EMB) had no available gene expression data. We followed the same procedure mentioned above for the remaining 65 groups to obtain the Spearman correlation between a group’s average gene expression and summary state assignment probabilities for the 1_TssA state.

We used a paired t-test to compare the correlations resulting from CSREP against those from base_count, with the alternative hypothesis that CSREP’s correlations with gene expression are higher than base_count’s.

**Applications of existing methods for detection of differential chromatin state domains across two sample groups**

In this section, we denote a *chromatin state* as the learned states that represents the combinatorial patterns of chromatin mark signals, outputted by methods such as ChromHMM and Segway (Libbrecht *et al.*, 2021, Ernst and Kellis, 2010, 2012, Hoffman et al., 2012). A *chromatin state annotation* then denotes the assignment of chromatin states (e.g. active promoter, enhancer, quiescent states, etc.) to each *genomic bin (*or equivalently*, genomic position*), which are outputted by methods that discover chromatin states and conduct genome segmentation and annotation (e.g. ChromHMM and Segway) (Libbrecht *et al.*, 2021, Ernst and Kellis, 2010, 2012, Hoffman et al., 2012). Additionally, a *genomic region* or *domain* denotes a window along the genome that can span one or multiple genomic bins for which the chromatin state segmentation and annotation is defined. We note that CSREP is designed to output one differential score with respect to each chromatin state $s$ at each genomic bin $i$. CSREP generates output genome-wide instead of at specific user-defined genomic region. The output of CSREP, therefore, is a matrix of size $G\times S$, where $S$ denotes the total number of chromatin states and $G$ denotes the total number of bins in the genome.

Similar to CSREP, the difference between base_count’s summary chromatin state maps for two groups of samples can be used to calculate the base_count’s differential chromatin scores, to compare against those generated by CSREP. In addition, we compared CSREP’s differential chromatin state scores to three additional differential scores based on the approaches used from existing methods: SCIDDO, ChromDiff, and EpiCompare (Ebert and Schulz, 2021; Yen and Kellis, 2015; He and Wang, 2017) (see below). We decided not to apply EpiAlign or chromSwitch to compare against CSREP since these methods are intended for cases where users are interested in measuring the differential chromatin state maps at a particular broad region of the genome (spanning multiple bins of chromatin state assignments) (Ge *et al.*, 2019; Jessa and Kleinman, 2018). Meanwhile, CSREP scores the genome-wide differential chromatin state maps at the same resolution as the input annotations (e.g. 200bp bins here). We did not compare CSREP against dPCA (Ji *et al.*, 2013) since it aims at scoring genomic regions’ differential epigenetic patterns directly from the chromatin signal data, while CSREP aims to uncover differential chromatin domains from input chromatin state maps.

***SCIDDO***

To run SCIDDO, we followed the tutorial provided by the author on Github https://github.com/ptrebert/sciddo/blob/master/testdata/tutorial.md (Ebert and Schulz, 2021), and generated a list of differential chromatin domains between two conditions, where each domain can be one genomic bin of chromatin state (200-bp bin) or multiple bins. SCIDDO’s output contained overlapping differential chromatin domains with different values of SCIDDO scores. For each genomic bin, we averaged the SCIDDO differential scores across overlapping differential chromatin domains, and assigned 0 to genomic bins not reported in SCIDDO’s output, implying no differential signals in chromatin states between the two groups.

***ChromDiff***

ChromDiff’s provided implementation is specifically designed to calculate differential scores for annotated genes (Yen and Kellis, 2015). Therefore, we could not directly use ChromDiff software to obtain differential scores at the same resolution as CSREP (200bp in all presented analyses). Instead, we directly implemented the same statistical test as used in the ChromDiff paper, the Mann-Whitney U-test, to determine differences in the number of samples from each group being annotated as a state $s$ at each genomic position $i$ (Yen and Kellis, 2015). For example, if 3 out of 5 samples in group 1 and 0 out of 7 samples in group 2 are annotated as state $s$ at position $i$, then we applied the Mann-Whitney U test with two input vectors $\left[ 1,1,1,0,0 \right]$ and [$0, 0, 0, 0, 0, 0, 0]$ for state $s$ at position $i.$  The test was implemented using Python’s scipy package, and the alternative hypothesis (one-sided vs. two-sided test) was set based on the analysis purpose (see sections below about evaluating CSREP’s differential scores in various analyses). The statistical test’s output p-values of 1.0 imply no difference and of 0.0 imply highest difference between two groups. We converted such p-values into differential score by the function$score=1-p_{value},$ so that the scores are bounded $[0,1]$, with higher values implying greater differences across the two groups. The output of ChromDiff is a matrix of size $G\times S$, where $G$ is the total number of genomic bins, and $S$is the number of chromatin states.

***EpiCompare***

As EpiCompare (He and Wang, 2017) only supports comparisons of specific groups of states (enhancer and promoter state groups only), we could not directly compare CSREP with EpiCompare. However, we did reimplement the Fisher’s exact test, which is a statistical test supported by EpiCompare. Specifically, for each genomic bin $i$ and chromatin state $s$, a contingency table is constructed indicating the number of samples from each group that are annotated as state$s$ (or not as state $s$). We then applied Fisher’s exact test using Python’s scipy package to evaluate the significance of differential annotations between the two groups. The alternative hypothesis to the Fisher’s exact test was set based on the application purpose (see sections below about evaluating CSREP’s differential scores in various analyses). The differential scores were also obtained by the function $score=1-p_{value}$, to ensure that higher scores imply higher levels of difference across the two groups.

***Evaluating CSREP’s differential chromatin state maps between Male and Female groups in recovering chromosome X- associated genomic regions***

For evaluating chromatin state differences between Male and Female groups with respect to chromosome X and autosomes, we obtained data for samples whose sex is annotated as either Male or Female (not ‘Unknown’ or ‘Mixed’), according to the provided metadata for the samples with 18-state chromatin state maps from Roadmap Epigenomic Project (Roadmap Epigenomics Consortium *et al.*, 2015). In total, there are 44 Male samples and 25 Female samples. We then generated 30 sets of 3 Male samples (randomly chosen from 44 Male samples) and 3 Female samples (randomly chosen from 25 Female samples). We calculated the differential chromatin scores for the 30 sets of samples using CSREP, base_count and SCIDDO, Mann-Whitney U test (based on ChromDiff) and Fisher’s exact test (based on EpiCompare) (see *Applications of existing methods for detection of differential chromatin state domains across two sample groups*). For each set of input samples from Male and Female groups, we used the *two-sided* statistical tests for ChromDiff and EpiCompare, and obtained the *absolute values* of CSREP and base_count differential chromatin scores. Then, we obtained a list of annotated TSSs for *protein-coding* genes from the accompanying metadata of genes’ coordinates and strand provided by the Roadmap Epigenomics project. For each set of 3 Male and 3 Female samples, we obtained the differential chromatin scores from each method, as outlined above, for regions that overlap these TSSs, and divided the score range window into 100 equal-width bins. The score ranges from CSREP, base_count, Mann-Whitney U test and Fisher’s exact test is [0, 1] and from SCIDDO is $[0, maximum value]$. We then applied the same procedure as outlined in the above section (titled ‘*Calculating the ROC curves of prediction for a single chromatin state’s location*’) to obtain true-/false- positive rates and AUROCs in predictions of TSS-overlapping regions on chromosome X, among all genomic regions overlapping annotated TSSs on the autosomes and chrX. We repeated the same analysis for a total of 30 sets of $n$ Male and $n$ Female samples, with $n \in\{3,5,9,12,15\}.$ We note that not all 30 rounds of application of SCIDDO to calculate differential chromatin scores ran successfully, due to software failure. In particular, all applications of SCIDDO with 30 input sets of 15 Male and 15 Female samples $(n=15)$ failed. For such cases, we report the average AUROCs for only successful runs of SCIDDO (**Fig. 3B**).

***Evaluating CSREP’s differential chromatin state map in recovering regions associated with differential chromatin mark signals***

To evaluate recovering differential chromatin mark signals, we first downloaded the available broad peaks of DNase, H3K9ac and H3K27ac for samples from the ESC and Brain groups from Roadmap Epigenomics Project at https://egg2.wustl.edu/roadmap/data/byFileType/peaks/consolidated/broadPeak. A full list of links to data used in this analysis is provided in **Additional File 2**. For each of the three chromatin marks and each cell group (ESC or Brain), we used $bedtools intersect$ (Quinlan and Hall, 2010) to obtain a set of peaks that are shared across all samples in the respective cell group. We then used $bedtools subtract$ function to derive peaks that are present in ESC samples and missing in Brain samples, and vice versa. We treated these ESC-specific, Brain-specific chromatin peaks as the ground-truth for this analysis. The number of base pairs overlapping peaks for each group range from 2,735,377 bp (ESC-specific H3K9ac peaks) to 85,995,111 bp (Brain-specific H3K27ac peaks) (**Additional File 2**).

We generated differential chromatin state scores from CSREP and base_count for the ESC and Brain groups for Roadmap Epigenomics, by subtracting their probabilistic chromatin state predictions for Brain from those for ESC. We also applied the Mann-Whitney U test (based on ChromDiff) and Fisher’s exact test (based on EpiCompare) for the two groups of samples for each chromatin state (see section *Applications of existing methods for detection of differential chromatin state domains across two sample groups*)*.* For the task of predicting ESC-specific peaks, Mann-Whitney U test (ChromDiff) and Fisher’s Exact test (EpiCompare) were applied such that ESC samples were used as the foreground (first) sample set, and Brain samples as the background (second) set. The foreground and background sample sets were reversed for the task of predicting Brain-specific peaks. P-values were obtained from these two methods using one-sided test, with the alternative hypothesis that a state $s$ is more likely to be annotated at position $i$ in the foreground samples than in the background samples. The differential scores Mann-Whitney U test (ChromDiff) and Fisher Exact test (EpiCompare) were converted as $score=1-pValue$, so that higher score (bounded in $[0,1]$) implies higher magnitude of difference between the two groups of samples. The differential chromatin score matrices from CSREP, base_count, Mann-Whitney U test (ChromDiff) and Fisher’s exact test (EpiCompare) methods are of size $G\times S$, and denoted $D_{CSREP}$, $D_{BC}, D_{MannW}$ and $D_{Fisher}$, respectively, where  $D_{CSREP,i, s}$, $D_{BC, i,s}$, $D_{MannW,i, s}$, $D_{Fisher,i, s}$ denote the corresponding methods’ differential score for state $s$ at genomic position $i$, respectively. We also obtained SCIDDO scores, which measure genome-wide differential chromatin patterns between the two groups. We denote the genome-wide SCIDDO score vector as$D_{SCIDDO}$, and $D_{SCIDDO, i}$ as the score at genomic position $i$.

To use $D_{CSREP}$ or $D_{BC}$ to calculate the ROC for genome-wide prediction of bases associated with ESC-specific or Brain-specific chromatin marks’ peaks, we divided the score range window $\left[ -1, 1 \right]$ into 200 equal-width bins with lower bounds $l \in\{-1, -0.99, ..., 0.99\}$. To calculate ROCs for predicting bases in ESC-specific peaks, for each state $s$ and each differential score lower-bound $l$, we defined genomic positions where the differential scores for state $s$ being greater than or equal to $l$, denoted $\left\{ i:D_{--, i, s}\geq l \right\}$, as *predicted differential peaks*. We compared such predictions with the ground-truth peaks described above to obtain the true- and false-positive rates of prediction for state $s$. To calculate ROCs for predicting bases associated with DNase/H3K9ac/H3K27ac Brain-specific peaks, first, we reversed the sign of  $D_{CSREP}$and $D_{BC}$, resulting in differential score matrices where positive values for state $s$ at position $i$ implies that the respective position $(i)$ has a higher probability of being in state $s$ in Brain compared to ESC. Then, we applied a similar procedure as outlined above to calculate CSREP’s and base_count’s ROCs.

We applied a similar procedure as outlined above for CSREP and base_count to calculate ROC curves based on $D_{MannW}$, $D_{Fisher}$ and $D_{SCIDDO}$, except using different ranges of differential scores other than $[-1, 1].$ For Mann-Whitney U test and Fischer’s exact test, the range was$[0,1]$. For SCIDDO, the scores range was based on the observed minimum and maximum scores across the genome. We then applied the same procedure as for CSREP and base_count scores in each state to obtain the ROCs, as mentioned above.

**Supplementary Figure 1: Effects of varying genome proportion used for training in CSREP on accuracy and runtime.** We conducted leave-one-out analysis to evaluate CSREP’s accuracy in predicting a held-out sample’s chromatin state map, given varying fractions of the genome used for training (**Supp. Methods**). We applied the procedure on data from Roadmap Epigenomics, and reported the AUROC for 3 and 12 samples from Heart **(A)** and Blood **(B)** groups, respectively. The empirical runtime for CSREP to generate the summary chromatin state maps for a group of 2 Heart samples or 11 Blood samples in the leave-one-out analysis are reported in **(C)**.

**Supplementary Figure 2:** **Visualization of ESC and Brain sample’s input chromatin state maps and CSREP’s output for region chr5:156,012,600-156,022,400, hg19.** All the sections of tracks are annotated in the legend on the left. The first section of tracks shows the universal chromatin state annotation that can annotate the epigenome across cell types, with states EnhA6 and EnhWk4 previously characterized as active and weak enhancer states specifically in the Brain/neuron, respectively (Vu and Ernst, 2022). The following three sections of tracks show the CSREP summary state assignment probabilities for the Brain group, the CSREP summary state annotation for Brain group, and the Brain input samples’ chromatin state maps from Roadmap Epigenomics. The last four sections of tracks show ESC input samples’ chromatin state maps from Roadmap Epigenomics, CSREP summary state annotation and assignment probabilities for the ESC group and the differential scores of ESC-Brain annotations. This figure shows input and CSREP output data for a similar genomic region as in **Fig. 1C**.

**Supplementary Figure 3: Empirical run time of CSREP for summarizing chromatin annotations from 11 groups (of 64 samples in total) from Roadmap.** We ran CSREP on the high-performance computing cluster, where each job was allocated 4 cores with 4GB of memory per core. snakemake (Köster and Rahmann, 2012; Mölder *et al.*, 2021) parallelizes the steps in input data preprocessing across all 64 input samples. Additionally, snakemake parallelizes the training process to predict chromatin state maps in individual samples in each group. The total runtime includes data preprocessing time shared across all groups of samples (~42 minutes), and prediction time that is specific to each group and denoted in the table. The prediction run time reported in the table include (1) the maximum time span of one job that outputs predictions for one sample, out of all samples in each group, and (2) the time span for averaging the predictions across samples, to obtain the group-wide summary chromatin state maps.

**Supplementary Figure 4: Visualization of CSREP’s input and output data for an arbitrary 500-kb genomic window (chr5: 42,821,109-43,321,109, hg19).** The visualization shows CSREP’s strong agreement with the chromatin state maps from 10 input samples from Digestive and 3 samples from Heart tissue groups from Roadmap Epigenomics. In each panel, the first track shows the summary chromatin state map based on CSREP. The following 3 (Heart) and 10 (Digestive) tracks show input samples’ chromatin state maps. States are colored based on legend on the left. In the following 18 tracks, each track shows the probabilities of assignment for one of 18 states. This region is the same as in **Fig. 2A**.

**Supplementary Figure 5**: **Visualization of CSREP’s input and output data for an arbitrary 500-kb genomic window (chr12:79,237,500-79,737,500, hg19).** Similar to **Supp. Fig. 4**, for genomic region chr12:79,237,500-79,737,500, hg19.

**Supplementary Figure 6**: **Visualization of CSREP’s input and output data for an arbitrary 500-kb genomic window (chr10:2,290,673-2,790,673, hg19).** Similar to **Supp. Fig. 4**, for genomic region chr10:2,290,673-2,790,673, hg19.

**Supplementary Figure 7**: **Visualization of CSREP’s input and output data for an arbitrary 500-kb genomic window (chr2:109,461,695-109,961,695, hg19)**. Similar to **Supp. Fig. 4**, for genomic region chr2:109,461,695-109,961,695, hg19.

**Supplementary Figure 8: Visualization of CSREP’s input and output data for a genomic window overlapping the LGALS4 gene (chr19:39,292,311-39,303,740, hg19).** Gene LGALS4 shows the distinctly higher expression in cell types of the Digestive system, with gene expression profile across cell types in **Supp. Fig. 10**. The visualization shows UCSC Genome browser view of CSREP’s output summary chromatin state maps for 10 input samples from Digestive and 3 samples from Heart tissue groups from Roadmap Epigenomics Consortium. The first 18 tracks show the summary probability assignment of chromatin states for Heart samples, based on CSREP. The following track shows the summary chromatin state annotation for Heart samples. The following 3 (Heart) and 10 (Digestive) tracks show input samples’ chromatin state maps. The following tracks show the summary chromatin state map for sample of the Digestive cell groups, followed by the individual states’ summary state assignment probabilities. States are colored based on legend on the left.

**Supplementary Figure 9: Visualization of CSREP’s input and output data for a genomic window overlapping the MT3 gene (chr16:56,623,267-56,625,000, hg19).** Gene MT3 shows the distinctly higher expression in Brain cell types, with gene expression profile across cell types in **Supp. Fig. 10**. The visualization shows UCSC Genome browser view of CSREP’s output summary chromatin state maps for 12 input samples from Blood and 7 samples from Brain tissue groups from Roadmap Epigenomics. The first 18 tracks show the summary probability assignment of chromatin states for Blood samples, based on CSREP. The following track shows the summary chromatin state annotation for Blood samples. The following 12 (Blood) and 7 (Brain) tracks show input samples’ chromatin state maps. The following tracks show the summary chromatin state map for sample of the Brain cell groups, followed by the individual states’ summary state assignment probabilities. States are colored based on legend on the left.


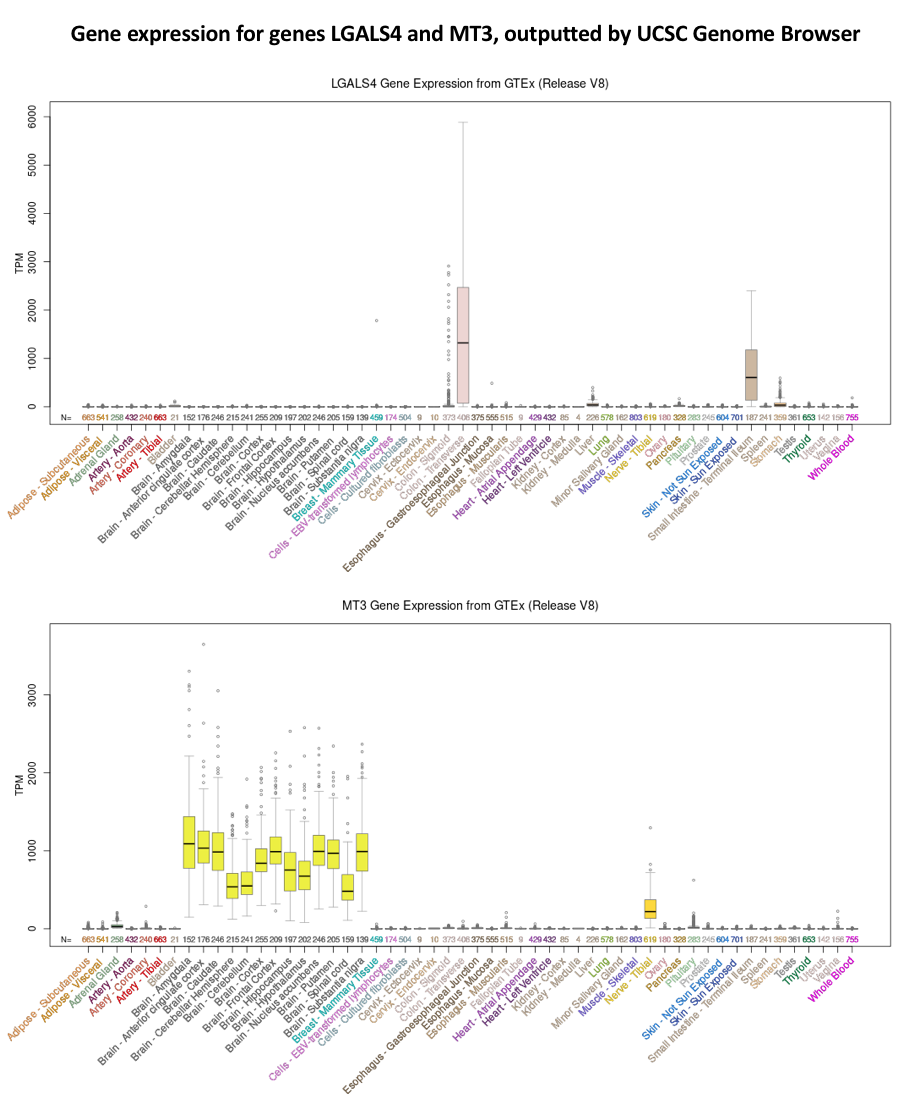


**Supplementary Figure 10: Gene expression profile for genes LGALS4 (top) and MT3 (bottom), as shown on UCSC Genome Browser.**

**Supplementary Figure 11:** **Relationship between the number of samples and AUROCs from using summary chromatin state map to predict genomic locations of individual chromatin states**. We conducted cross-validation analysis for each group of samples (**Supp. Methods**), and for each group, we calculate ROC curve of CSREP’s summary probabilistic chromatin state map in recovering genomic positions of individual chromatin states in a held-out sample. Each panel corresponds to a chromatin state, and shows the difference between CSREP’s AUROCs and base_count’s AUROCs for predicting locations of the chromatin state in left-out samples. Each dot corresponds to one sample. Y-axis shows the difference of AUROCs between the two methods (positive y-axis means CSREP results in higher AUROCs compared to base_count, and vice-versa). X-axis shows the number of input samples for the group, not to scale, but the reported Pearson correlation is based on actual number of samples.

**Supplementary Figure** **12**: **Histogram of CSREP differential chromatin scores between Male and Female groups of samples, in autosomes and in chromosome X.** Each subpanel shows the histograms of one state’s CSREP *Male - Female* differential scores, bounded between -1 and 1, in autosomes and chromosome X.

**Supplementary Figure 13:** **Mean and variance of the CSREP differential scores between Male and Female groups of samples, in autosomes and in chromosomes.** The mean differential scores for each state in either chromosome X or autosomes are reported for each state and share the same color scale as in bottom legend. The difference between the mean scores for chromosome X and the autosomes for each state is reported on the bottom row and colored as in bottom legend. The three states with largest-magnitude difference in mean scores are 13_Het, 17_ReprPCWk, 18_Quies.

**Supplementary Figure 14: Evaluation of recovery of differential chromatin marks signals between ESC and Brain.** The table is an extension to **Fig. 4**, and shows AUROCs for differential scores’ predictions of genomic regions associated with differential peak signals for one chromatin mark, from left to right: DNase, H3K27ac and H3K9ac. For each chromatin mark, it shows the AUROCs of predicting signal peaks observed in Brain and ESC exclusively (Brain-spec and ESC-spec). Differential scores outputted by CSREP, base-count, Mann-Whitney U test (used by ChromDiff) and Fisher’s exact test (used by EpiCompare) are shown for each chromatin state (rows). In each category of comparisons (a chromatin mark in either ESC or Brain), the top three scores that show highest AUROCs are highlighted in green. Along the bottom is the AUROC for SCIDDO. The differential scores for states that are not related to active promoter and enhancer activities tend to show AUROCs near or lower than 0.5, which is expected since these states are not associated with DNase, H3K27ac or H3K9ac.

**References**

Boix,C.A. *et al.* (2021) Regulatory genomic circuitry of human disease loci by integrative epigenomics. *Nature*, **590**, 300–307.

Ebert,P. and Schulz,M.H. (2021) Fast detection of differential chromatin domains with SCIDDO. *Bioinformatics*, **37**, 1198–1205.

Ge,X. *et al.* (2019) EpiAlign: an alignment-based bioinformatic tool for comparing chromatin state sequences. *Nucleic Acids Res.*, **47**, e77–e77.

He,Y. and Wang,T. (2017) EpiCompare: an online tool to define and explore genomic regions with tissue or cell type-specific epigenomic features. *Bioinformatics*, **33**, 3268–3275.

Jessa,S. and Kleinman,C.L. (2018) Chromswitch: a flexible method to detect chromatin state switches. *Bioinformatics*, **34**, 2286–2288.

Ji,H. *et al.* (2013) Differential principal component analysis of ChIP-seq. *Proc. Natl. Acad. Sci.*, **110**, 6789–6794.

Köster,J. and Rahmann,S. (2012) Snakemake—a scalable bioinformatics workflow engine. *Bioinformatics*, **28**, 2520–2522.

Libbrecht,M.W. *et al.* (2021) Segmentation and genome annotation algorithms for identifying chromatin state and other genomic patterns. *PLoS Comput. Biol.*, **17**, e1009423.

Mölder,F. *et al.* (2021) Sustainable data analysis with Snakemake. *F1000Research*, **10**.

Quinlan,A.R. and Hall,I.M. (2010) BEDTools: a flexible suite of utilities for comparing genomic features. *Bioinformatics*, **26**, 841–842.

Roadmap Epigenomics Consortium *et al.* (2015) Integrative analysis of 111 reference human epigenomes. *Nature*, **518**, 317–330.

Vu,H. and Ernst,J. (2022) Universal annotation of the human genome through integration of over a thousand epigenomic datasets. *Genome Biol.*, **23**, 1–37.

Yen,A. and Kellis,M. (2015) Systematic chromatin state comparison of epigenomes associated with diverse properties including sex and tissue type. *Nat. Commun.*, **6**, 1–13.
